# Supplementary material for: Cross-GWAS coherence test at the gene and pathway level
Source: PLoS Comput Biol. 2022 Sep 26;18(9):e1010517. doi: 10.1371/journal.pcbi.1010517 (PMC9536597; doi:10.1371/journal.pcbi.1010517)
Supplement: S1 Text — (PDF) [file pcbi.1010517.s001.pdf]

September 2022

Cross-GWAS coherence test at the gene and pathway level: Supplementary Text

Daniel Krefl[1,2,*] and Sven Bergmann[1,2,3,*]
[1] *Department of Computational Biology, University of Lausanne, Switzerland*
[2] *Swiss Institute of Bioinformatics, Lausanne, Switzerland*
[3] *Dept. of Integrative Biomedical Sciences, University of Cape Town, Cape Town, South Africa*
[*] *daniel.krefl@unil.ch (DK);* [*] *sven.bergmann@unil.ch (SB)*

1. GENERAL PRODUCT-NORMAL

Let us briefly consider the distribution for xy with $x \sim \mathcal{N}(\mu_x, \sigma_x^2)$ and $y \sim \mathcal{N}(\mu_y, \sigma_y^2)$, *i.e.*, the product-normal for non-standardized Gaussian random variables. The moment generating function reads [1]

$$M_{x,y}(\nu) = \frac{e^{\frac{(\mu_x^2\sigma_y^2+\mu_y^2\sigma_x^2-2\varrho\mu_x\mu_y\sigma_x\sigma_y)\nu^2+2\mu_x\mu_y\nu}{2(1-\sigma_x\sigma_y(1-\varrho)\nu)(1+\sigma_x\sigma_y(1+\varrho)\nu)}}}{\sqrt{(1-\sigma_x\sigma_y(1+\varrho)\nu)(1+\sigma_x\sigma_y(1-\varrho)\nu)}}\,. \tag{1}$$

Note that for, either, $\mu_x = 0$ or $\mu_y = 0$, the factorization of the main text still holds. Defining $\kappa_x = \frac{\mu_x}{\sigma_x}$ and $\kappa_y = \frac{\mu_y}{\sigma_y}$, the exponent of the exponential in $M_{x,y}\left(\frac{2\nu}{\sigma_x\sigma_y}\right)$ can be split for the general non-correlated case ($\varrho = 0$) into

$$\frac{(\kappa_x^2+\kappa_y^2+2\kappa_x\kappa_y)\nu}{2(1-2\nu)} - \frac{(\kappa_x^2+\kappa_y^2-2\kappa_x\kappa_y)\nu}{2(1+2\nu)}\,.$$

We deduce that we can still factorize the moment generating function, such that

$$\begin{aligned} xy \sim & \frac{\sigma_x\sigma_y}{2}\left[\chi_1^2\left(\frac{1}{2}(\kappa_x^2+\kappa_y^2+2\kappa_x\kappa_y)\right)\right] \\ & - \frac{\sigma_x\sigma_y}{2}\left[\chi_1^2\left(\frac{1}{2}(\kappa_x^2+\kappa_y^2-2\kappa_x\kappa_y)\right)\right]\,, \end{aligned} \tag{2}$$

with $\chi_1^2(c)$ the non-central χ^2 distribution with one degree of freedom. Hence, the general product-normal can be expressed as a linear combination of non-central χ_1^2 distributions, for $\varrho = 0$.

2. PROBABILITY DENSITY FUNCTIONS

a. Product-Normal: Making use of the relation (2) for standardized variables and expressed in terms of gamma distributions, the pdf, denoted as f, of the product-normal distribution can be calculated analytically via convolution (*cf.*, [2])

$$f_{\xi-\zeta}(x) = \frac{e^{\frac{x}{1-\varrho}}}{\pi\sqrt{1-\varrho^2}}\int_{\max(0,x)}^{\infty} dy (y^2-xy)^{-1/2} e^{-\frac{2y}{1-\varrho^2}}\,.$$

ϱ	0	0.3	0.6	0.9
MSE	3.3×10^{-15}	3.5×10^{-15}	4.6×10^{-15}	2.1×10^{-12}

Table 1: Mean squared error (MSE) between numerical integration of (3) and Davies algorithm for various ϱ and 1000 arguments evenly spaced in the range $[-4, 4]$.

Completing the square and invoking a hyperbolic substitution, we arrive at

$$\begin{aligned} f_{\xi-\zeta}(x) &= \frac{e^{\frac{\varrho x}{1-\varrho^2}}}{\pi\sqrt{1-\varrho^2}}\int_0^{\infty} dt\, e^{-\frac{|x|}{1-\varrho^2}\cosh(t)} \\ &= \frac{e^{\frac{\varrho x}{1-\varrho^2}}}{\pi\sqrt{1-\varrho^2}} K_0\left(\frac{|x|}{1-\varrho^2}\right)\,, \end{aligned} \tag{3}$$

with K_0 the modified Bessel function of the second kind at zero order. The result above for f agrees with the previous derivations of [3, 4]. Note that the analytic calculation of the corresponding cdf requires the solution of an integral of the type $\int_x^{\infty} dt\, e^{at}K_0(t)$. We are not aware of a known closed-form solution.

For illustration, we plot the pdf for $\varrho = 1/2$ together with the corresponding histogram sampled from (2) expressed as a difference of gamma distributions in Fig 1. We also show the cdf obtained via numerical integration of (3). We verified that the numerical integration matches the results obtained via Davies' algorithm for the cdf calculation for various ϱ, *cf.*, Table 1.

Note that since the pdf of the non-central χ^2 distribution includes a Bessel function, analytic calculation of the pdf of xy in the more general case of section 1 is more complicated than in (3), and will not be discussed here.

b. Variance-Gamma: Consider a random variable X distributed according to

$$X \sim [\Gamma(h/2, g)] - [\Gamma(h/2, g)] =: VG(h, g)\,. \tag{4}$$

The corresponding pdf can be calculated similarly as above via convolution. We infer

$$f_{\xi-\zeta}(x) = \frac{e^{\frac{x}{g}}}{\Gamma(h/2)^2 g^h}\int_{\max(0,x)}^{\infty} dy\,(y^2-xy)^{h/2-1} e^{-\frac{2y}{g}}\,.$$

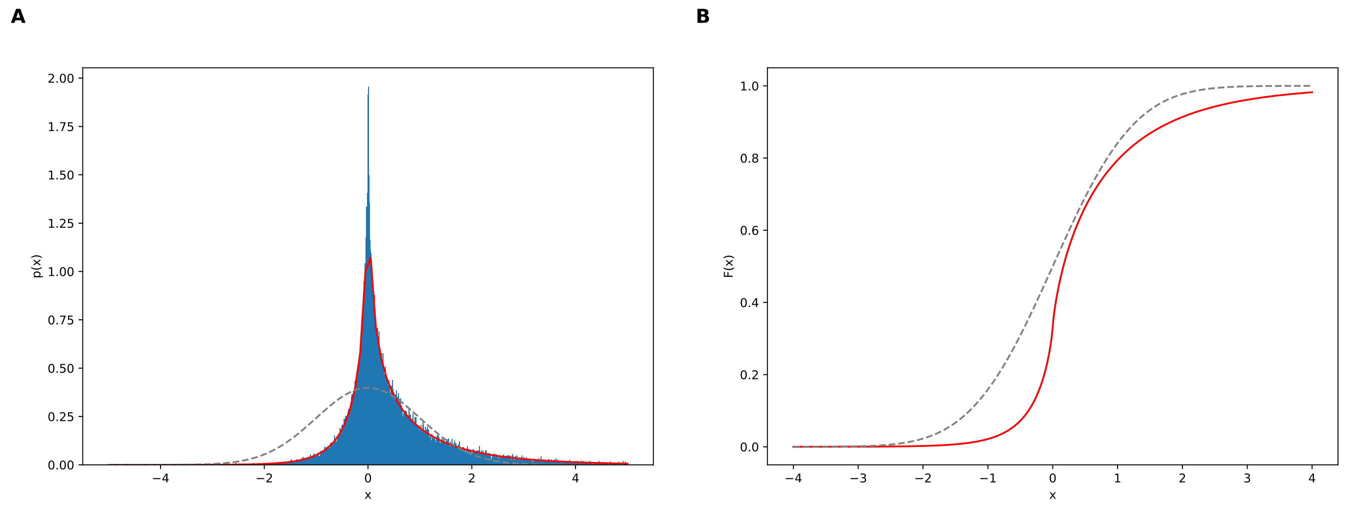

Fig 1: A: Histogram of the dependent product normal distribution with $\rho = 0.5$ obtained via subtracting 50.000 pairs of random samples of difference of gamma distributions. The red line marks the pdf given in (3). B: The corresponding cdf obtained via Davies algorithm. For comparison, the dashed gray lines show the corresponding normal quantities.

Completing the square and using as before hyperbolic substitution, we arrive at

$$\begin{aligned} f_{\xi-\zeta}(x) &= \frac{x^{h-1}}{2^{h-1}\Gamma(h/2)^2 g^h}\int_0^\infty dt\, \sinh(t)^{h-1} e^{-\frac{|x|}{g}\cosh(t)} \\ &= \frac{K_{\frac{h-1}{2}}(|x|/g)}{g\sqrt{\pi}\,\Gamma(h/2)}\left(\frac{|x|}{2g}\right)^{\frac{h-1}{2}}, \end{aligned} \tag{5}$$

with K_n a modified Bessel function of second kind at order n. Using the integral $\int_0^\infty dt\, t^{\mu-1}K_\nu(t) = 2^{\mu-2}\Gamma(\frac{\mu-\nu}{2})\Gamma(\frac{\mu+\nu}{2})$, we easily verify that $\int_0^\infty dx\, f_{\xi-\zeta}(x) = \frac{1}{2}$. Hence, due to symmetry the pdf is well normalized. However, we are not aware of closed-form solutions for Bessel function integrals of the type $\int_x^\infty dt\, t^\nu K_\nu(t)$, which are needed to provide a closed-form expression for the cdf.

The distribution given by (5) occurred before in the finance domain as a special case of the *variance-gamma* distribution [5]. It can be traced back further to the distribution of the bivariate correlation. In detail, the gamma-variance distribution corresponds to the off-diagonal marginal of a two-dimensional Wishart distribution, which models the covariance matrix [6]. However, to the best of our knowledge, what is new is the expression in terms of the difference distribution in equation (4).

For $h = n$ and $g = 1$, we have

$$X \sim VG(n,1) = \frac{1}{2}[\chi_n^2] - \frac{1}{2}[\chi_n^2]\,. \tag{6}$$

Hence, for $n = 1$, we obtain the product-normal distribution with $\varrho = 0$ as discussed in the previous section. For general n, we can view $VG(n,1)$ as the distribution of a sum of n independently distributed product-normal random variables. In particular, we can use Davies' algorithm to calculate the cdf for $VG(n,1)$ exactly at the desired precision.

3. SIMULATIONS: $N = 2$

The importance of correcting for the inter-dependence between the elements of w and z in the index $I = \sum_i w_i z_i$ introduced in the main text can be seen easily in the $N = 2$ case. Consider the covariance matrices,

$$\Sigma = \Sigma_w = \Sigma_z = \begin{pmatrix} 1 & r \\ r & 1 \end{pmatrix},$$

such that $w \sim \mathcal{N}(0,\Sigma)$ and $z \sim \mathcal{N}(0,\Sigma)$, and with r varying. In Fig 2 we show various significance threshold curves of I for varying r, as calculated from the to I corresponding distribution expressed as a linear combination of χ^2 distributions, *cf.*, the main text. Clearly, for increasing inter-element correlation, the significance threshold level rises. The magnitude of the effect increases with the desired level of significance.

4. ACCOUNTING FOR SAMPLE OVERLAP

For GWAS, the populations used to investigate two traits for co-significant signals might overlap. In particular, it may even be that the GWAS for the two traits have been conducted for the same population (full overlap). If

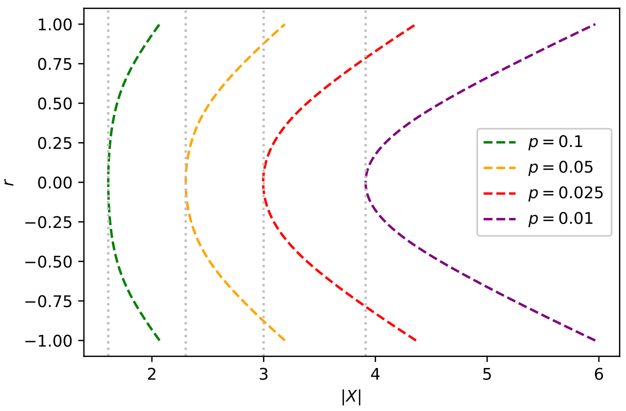

Fig 2: Significance threshold curves of I in the two-element case under variation of the inter-element correlation (y-axis). The x-axis corresponds to the argument of the tail probability $p = 1 - F_I(X)$, with F_I the cdf of the distribution of I. The gray dotted lines mark the minimal value obtained for zero correlation.

there is in addition a strong phenotypic correlation between the traits, a danger for a significant biased result is present, as long as one does not correct for this effect induced by the sample overlap.

Let us consider for simplicity first the case of full population sample overlap. For same population (and so genotype matrix X), and for two different traits $y^{(1)}$ and $y^{(2)}$, we have as multi-variate models

$$\begin{aligned} y^{(1)} &= X\alpha^{(1)} + \epsilon^{(1)}\,, \\ y^{(2)} &= X\alpha^{(2)} + \epsilon^{(2)}\,, \end{aligned} \tag{7}$$

with true effect sizes $\alpha^{(i)}$ and residuals $\epsilon^{(i)} \sim \mathcal{N}(0, \mathbb{1})$.

Our test null assumption is that $\alpha^{(i)} = 0$. Therefore,

$$\mathrm{cov}(y^{(1)}, y^{(2)}) = \mathrm{cov}(\epsilon^{(1)}, \epsilon^{(2)}) = (\epsilon^{(1)})^T \epsilon^{(2)} := \zeta\,.$$

We can assume that the residuals are independent for independent populations but generally $\zeta \neq 0$ for overlapping populations. This implies

$$\begin{pmatrix} \epsilon^{(1)} \\ \epsilon^{(2)} \end{pmatrix} \sim \mathcal{N}\left(0, \begin{pmatrix} \mathbb{1} & \zeta\mathbb{1} \\ \zeta\mathbb{1} & \mathbb{1} \end{pmatrix}\right)\,. \tag{8}$$

Recall from the main text that the z-scored null effect estimates read (as vector over some region)

$$z = \frac{1}{\sqrt{n}} x^T \epsilon \sim \mathcal{N}(0, \Sigma),$$

with $\Sigma := \frac{1}{n} x^T x$ and x_i columns of X. It follows that

$$\begin{pmatrix} z^{(1)} \\ z^{(2)} \end{pmatrix} = \frac{1}{\sqrt{n}} \begin{pmatrix} x^T & \\ & x^T \end{pmatrix} \begin{pmatrix} \epsilon^{(1)} \\ \epsilon^{(2)} \end{pmatrix} \sim \mathcal{N}\left(0, \begin{pmatrix} \Sigma & \zeta\Sigma \\ \zeta\Sigma & \Sigma \end{pmatrix}\right)\,. \tag{9}$$

Using block Gaussian elimination, one can show that

$$\begin{pmatrix} \Sigma & \zeta\Sigma \\ \zeta\Sigma & \Sigma \end{pmatrix} = \begin{pmatrix} \mathbb{1} & \\ \zeta\mathbb{1} & \mathbb{1} \end{pmatrix} \begin{pmatrix} \Sigma & \\ & (1-\zeta^2)\Sigma \end{pmatrix} \begin{pmatrix} \mathbb{1} & \zeta\mathbb{1} \\ & \mathbb{1} \end{pmatrix}\,. \tag{10}$$

Hence,

$$\begin{pmatrix} z^{(1)} \\ z^{(2)} \end{pmatrix} \sim \begin{pmatrix} \mathbb{1} & \\ \zeta\mathbb{1} & \mathbb{1} \end{pmatrix} \begin{pmatrix} x \\ y \end{pmatrix} = \begin{pmatrix} x \\ y + \zeta x \end{pmatrix}\,, \tag{11}$$

with

$$\begin{pmatrix} x \\ y \end{pmatrix} \sim \mathcal{N}\left(0, \begin{pmatrix} \Sigma & \\ & (1-\zeta^2)\Sigma \end{pmatrix}\right)\,. \tag{12}$$

We conclude that

$$\mathrm{cov}(z_i^{(1)}, z_j^{(2)}) = \mathrm{cov}(x_i, y_j + \zeta x_j) = \zeta\,\mathrm{cov}(x_i, x_j) = \zeta\Sigma_{ij}\,.$$

Consider now a matrix U such that $\Sigma = U\Lambda U^T$ with Λ the diagonal matrix of eigenvalues. Clearly, $UU^T = 1$. We then have

$$\begin{aligned} z^{(1)} &= U^T\sqrt{\Lambda}w \\ z^{(2)} &= U^T\sqrt{\Lambda}v \end{aligned} \tag{13}$$

with $w, v \sim \mathcal{N}(0, \mathbb{1})$. The correlation between the new variables w and v can be calculated to be given by

$$\begin{aligned} \mathrm{corr}(w_i, v_j) &= \frac{\mathrm{cov}(w_i, v_j)}{\sqrt{\mathrm{var}(w_i)}\sqrt{\mathrm{var}(v_j)}} \\ &= \frac{\mathrm{cov}(\frac{1}{\sqrt{\lambda_i}}(Uz^{(1)})_i, \frac{1}{\sqrt{\lambda_j}}(Uz^{(2)})_j)}{\sqrt{\mathrm{var}(\frac{1}{\sqrt{\lambda_i}}(Uz^{(1)})_i)}\sqrt{\mathrm{var}(\frac{1}{\sqrt{\lambda_j}}(Uz^{(2)})_j)}} \\ &= \frac{\sum_{k,l} U_{ik}^T U_{lj} \mathrm{cov}(z_k^{(1)}, z_l^{(2)})}{\sqrt{\mathrm{var}((Uz^{(1)})_i)}\sqrt{\mathrm{var}((Uz^{(2)})_j)}} \\ &= \frac{\sum_{k,l} U_{ik}^T U_{li} \mathrm{cov}(z_k^{(1)}, z_l^{(2)})}{\sqrt{\lambda_i\lambda_j}} \\ &= \zeta\frac{\sum_{k,l} U_{ik}^T \Sigma_{kl} U_{lj}}{\sqrt{\lambda_i\lambda_j}} \\ &= \zeta\frac{\Lambda_{ij}}{\sqrt{\lambda_i\lambda_j}}\,. \end{aligned} \tag{14}$$

It follows that

$$\mathrm{corr}(w_i, v_i) = \begin{cases} \zeta & i = j \\ 0 & i \neq j \end{cases}\,. \tag{15}$$

a. Coherence test Recall from the main text (Methods, *Product-Normal distribution*) that the product distribution for correlated $\mathcal{N}(0,1)$ variables with coefficient ϱ can be expressed as

$$w_i v_i \sim \frac{1+\varrho}{2}[\chi_1^2] - \frac{1-\varrho}{2}[\chi_1^2]\,.$$

We conclude that for the corrected null distribution with identical populations for the two traits

$$(z^{(1)})^T z^{(2)} \sim \sum_i \lambda_i \left(\frac{1+\zeta}{2}\right)[\chi_1^2] - \sum_i \lambda_i \left(\frac{1-\zeta}{2}\right)[\chi_1^2]. \tag{16}$$

The coefficient ζ is given by the phenotypic correlation. As one would expect, for identical traits ($\zeta = 1$), the above turns into the standard χ^2 test of Pascal [7]. For $\zeta = 0$, we end up with the formula (2.2) of the main text.

The generalization to partial sample overlap is straightforward. We can assume that the samples are sorted such that the overlapping samples form the first n_o rows, followed by the $n_i^{(x)}$ independent samples with $n_o + n_i^{(1)} + n_i^{(2)} = n$. Correspondingly, the residuals read

$$\begin{pmatrix} \epsilon^{(1)} \\ \epsilon^{(2)} \end{pmatrix} \sim \mathcal{N}\left(0, \begin{pmatrix} \mathbb{1}_{n_o\times n_o} & & \zeta\mathbb{1}_{n_o\times n_o} & \\ & \mathbb{1}_{n_i^{(1)}\times n_i^{(1)}} & & \\ \zeta\mathbb{1}_{n_o\times n_o} & & \mathbb{1}_{n_o\times n_o} & \\ & & & \mathbb{1}_{n_i^{(2)}\times n_i^{(2)}} \end{pmatrix}\right), \tag{17}$$

and the z-scored null effect estimates

$$\begin{pmatrix} z^{(1)} \\ z^{(2)} \end{pmatrix} = \begin{pmatrix} \frac{1}{\sqrt{n_o+n_i^{(1)}}}(x^{(1)})^T & \\ & \frac{1}{\sqrt{n_o+n_i^{(2)}}}(x^{(2)})^T \end{pmatrix} \begin{pmatrix} \epsilon^{(1)} \\ \epsilon^{(2)} \end{pmatrix}, \tag{18}$$

with the first n_o rows of $x^{(1)}$ identical to $x^{(2)}$.

We assume that the population covariance matrix Σ is well approximated by the sub-populations of both GWAS, *i.e.*,

$$\frac{1}{n_o + n_i^{(k)}}(x^{(k)})^T\, \mathbb{1}_{(n_o+n_i^{(k)})\times(n_o+n_i^{(k)})}\, x^{(k)} \approx \Sigma .$$

For the off-diagonal parts, we have

$$\frac{\left(x^{(k)}\right)^T \begin{pmatrix} \mathbb{1}_{n_o\times n_o} & \\ & \end{pmatrix} \left(x^{(l)}\right)}{\sqrt{n_i^{(1)} + n_o}\sqrt{n_i^{(2)} + n_o}} \approx \frac{\Sigma}{\sqrt{1+\frac{n_i^{(1)}}{n_o}}\sqrt{1+\frac{n_i^{(2)}}{n_o}}},$$

under the assumption that the overlapping sub-population also approximates the population covariance structure well.

It follows that the sample overlap calculation proceeds as for the full overlap case discussed above, but with

$$\zeta \to \frac{n_o}{\sqrt{n^{(1)}}\sqrt{n^{(2)}}}\,\zeta, \tag{19}$$

with $n^{(k)} = n_o + n_i^{(k)}$ the individual GWAS sample sizes. Note that for n_o sufficiently small the correction factor vanishes, as it should be.

The calculation of ζ requires knowledge of the phenotypic correlation. However, often only public GWAS summary statistics are at hand. Fortunately, in this case, one can use LD score regression [8] to obtain an estimation, as the intercept term thereof precisely corresponds to the correction factor (19).

In order to illustrate the impact of such a non-zero correction factor, we consider a multivariate normal distribution with correlation matrix Σ of dimension 100. Σ is taken to be glued together from two 50-dimensional correlation matrices with off-diagonal elements set identical to 0.2 and two off-diagonal block matrices with elements of 0.2 multiplied by various ζ. This setup simulates (9).

We calculate p-values for the index $(z^{(1)})^T z^{(2)}$ given in equation (16) for 1000 random samples with and without correction factor ζ. Corresponding QQ-plots are shown in Fig 3 and Fig 4. We observe that inclusion of ζ indeed removes the bias.

b. Ratio test The index of the ratio test reads (see Results, section *Ratio test* of the main text)

$$R = \frac{(z^{(1)})^T z^{(2)}}{(z^{(1)})^T z^{(1)}}, \tag{20}$$

with $z^{(i)}$ defined as above. (Similarly for interchanged indices.) We have

$$\begin{aligned} \Pr(R \le r) &= \Pr\left((z^{(1)})^T z^{(2)} \le r (z^{(1)})^T z^{(1)}\right) \\ &= \Pr\left((z^{(1)})^T (z^{(2)} - r\, z^{(1)}) \le 0\,.\right) \end{aligned} \tag{21}$$

We can linearly transform variables such that

$$\Pr(R \le r) = \Pr(w^T \Lambda (v - r\, w) \le 0).$$

Under the re-definition $u := v - r\, w$ we arrive at

$$\Pr(R \le r) = \Pr(w^T \Lambda u \le 0) = F_{\bar{w}\bar{u}}(0),$$

as in the main text. However, because of the non-vanishing correlation (15), we now have instead $\bar{w} \sim \mathcal{N}(0, \Lambda)$ and $\bar{u} \sim \mathcal{N}(0, (1 + r^2 - 2\zeta r)\Lambda)$. In particular,

$$\mathrm{cov}(w_i, u_j) = \mathrm{cov}(w_i, v_j) - r\delta_{ij} = (\zeta - r)\delta_{ij}.$$

With $\mathrm{Var}(w_i) = 1$ and $\mathrm{Var}(u_j) = (1 + r^2 - 2\zeta r)$ we obtain for the corresponding correlation

$$\mathrm{corr}(w_i, u_j) = \frac{(\zeta - r)\delta_{ij}}{\sqrt{(1 + r^2 - 2\zeta r)}}.$$

Therefore, we deduce similar as in the main text that

$$\begin{aligned} \bar{w}\bar{u} \sim \sum_i & \frac{\lambda_i\sqrt{1 + r^2 - 2\zeta r}(1+t)}{2}[\chi_1^2] \\ & - \frac{\lambda_i\sqrt{1 + r^2 - 2\zeta r}(1-t)}{2}[\chi_1^2], \end{aligned} \tag{22}$$

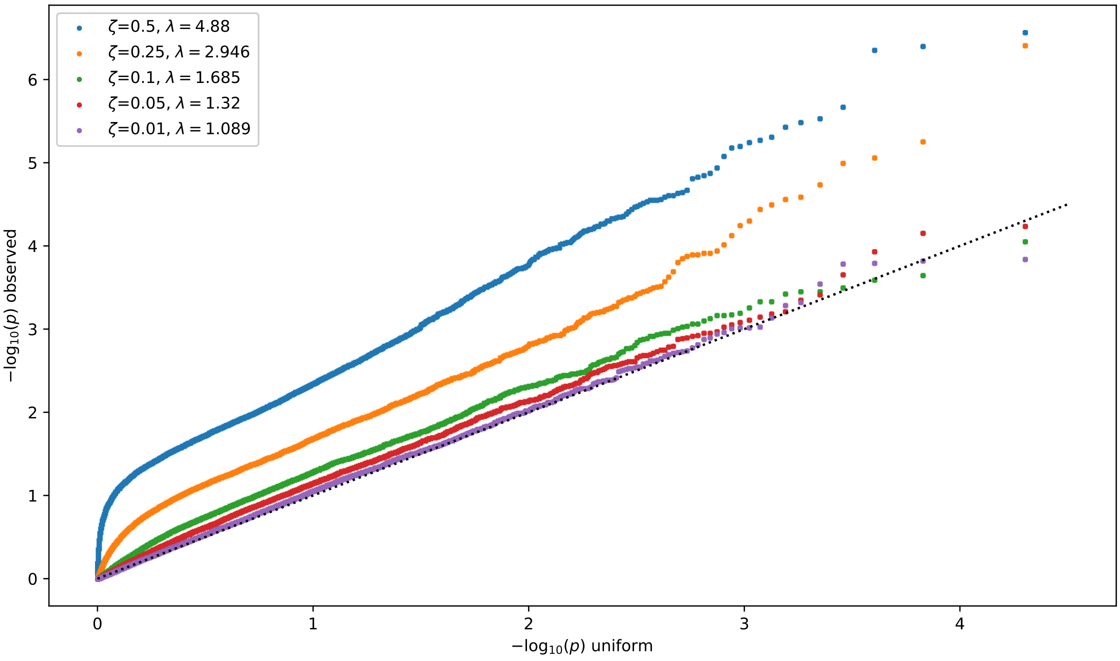

Fig 3: QQ plot for simulation of the index (16) without applied correction for ζ for various values of ζ. The control factor λ is taken to be the median of observed $-\log_{10}$ transformed p-values divided by $-\log_{10}(0.5)$. An observed $\lambda < 1.1$ is commonly viewed as acceptable.

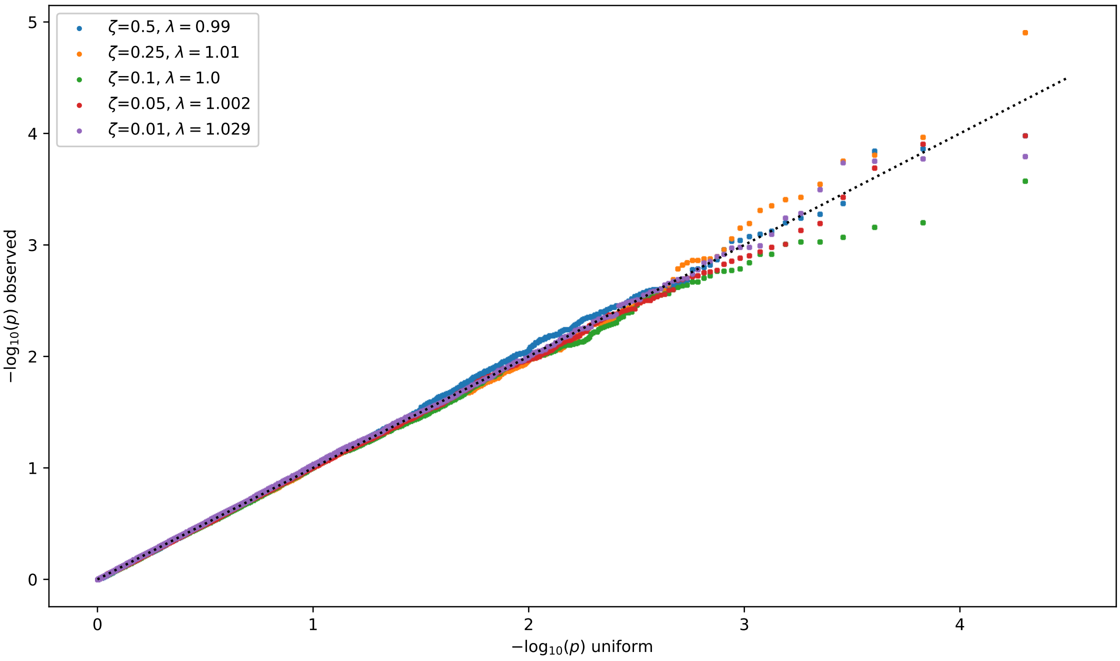

Fig 4: QQ plot for simulation of the index (16) with applied correction for ζ for same values of ζ as in Fig 3. λ is defined as in Fig 3. After applying the correction, the p-values are indeed well calibrated and λ close to one.

with

$$t := \frac{\zeta - r}{\sqrt{1 + r^2 - 2\zeta r}} \, .$$

Similar to the main text, a consistency check can be performed against the Cauchy distribution. We know that for two normal distributed random variables $x \sim \mathcal{N}(0, \sigma_x^2)$ and $y \sim \mathcal{N}(0, \sigma_y^2)$ with correlation ζ,

$$\frac{x}{y} \sim \mathcal{C}\left(\frac{\sigma_x}{\sigma_y}\zeta, \frac{\sigma_x}{\sigma_y}\sqrt{1-\zeta^2}\right) \, ,$$

with $\mathcal{C}$ the general Cauchy distribution. The corresponding cumulative distribution function for our case reads

$$F = \frac{1}{2} + \frac{1}{\pi}\arctan\left(\frac{r - \zeta}{\sqrt{1-\zeta^2}}\right) \, .$$

Explicit evaluation shows agreement with the χ^2 based calculation via equation (22).

For partial sample overlap, the previous discussion for the coherence test applies one to one to the ratio test, and therefore the phenotypic correlation ζ has to be adjusted as well according to equation (19). As for the coherence, we calculate p-values for the index R defined in equation (20) for 1000 random samples with and without correction factor ζ. Corresponding QQ-plots are shown in Fig 5 and Fig 6. We observe that the correction indeed improves the calibration of p-values.

References

1. Craig CC. On the frequency function of xy. Ann Math Stat. 7 (1936) 1-15.
2. Poe GL, Severance-Lossin EK, Welsh MP. Measuring the Difference (X - Y) of Simulated Distributions: A Convolutions Approach. American Journal of Agricultural Economics Vol. 76, No. 4 (Nov., 1994), pp. 904-915.
3. Nadarajah S, Pogany TK. On the distribution of the product of correlated normal random variables. C R Acad Sci Paris. Ser I 354 (2016) 201-204.
4. Cui G, Yu X, Iommelli S, Kong L. Exact Distribution for the Product of Two Correlated Gaussian Random Variables. IEEE Signal Processing Letters, 23(11), 1662–1666. doi.org/10.1109/lsp.2016.2614539
5. Dilip BM, Seneta E. The Variance Gamma (V.G.) Model for Share Market Returns The Journal of Business. Vol. 63, No. 4 (Oct., 1990), pp. 511-524 (14 pages).
6. Pearson K, Jeffery GB, Elderton EM. On the Distribution of the First Product Moment-Coefficient, in Samples Drawn from an Indefinitely Large Normal Population. Biometrika. Biometrika Trust. 21: 164–201, December 1929. doi.org/10.2307/2332556
7. Lamparter D, Marbach D, Rueedi R, Kutalik Z, Bergmann S. Fast and rigorous computation of gene and pathway scores from SNP-based summary statistics. PLoS Computational Biology 12, e1004714, 2016.
8. Bulik-Sullivan B, Finucane HK, Anttila V, Gusev A, Day FR, Loh PR, et al. An atlas of genetic correlations across human diseases and traits. Nat Genet. 2015;47(11):1236-1241. doi.org/10.1038/ng.3406

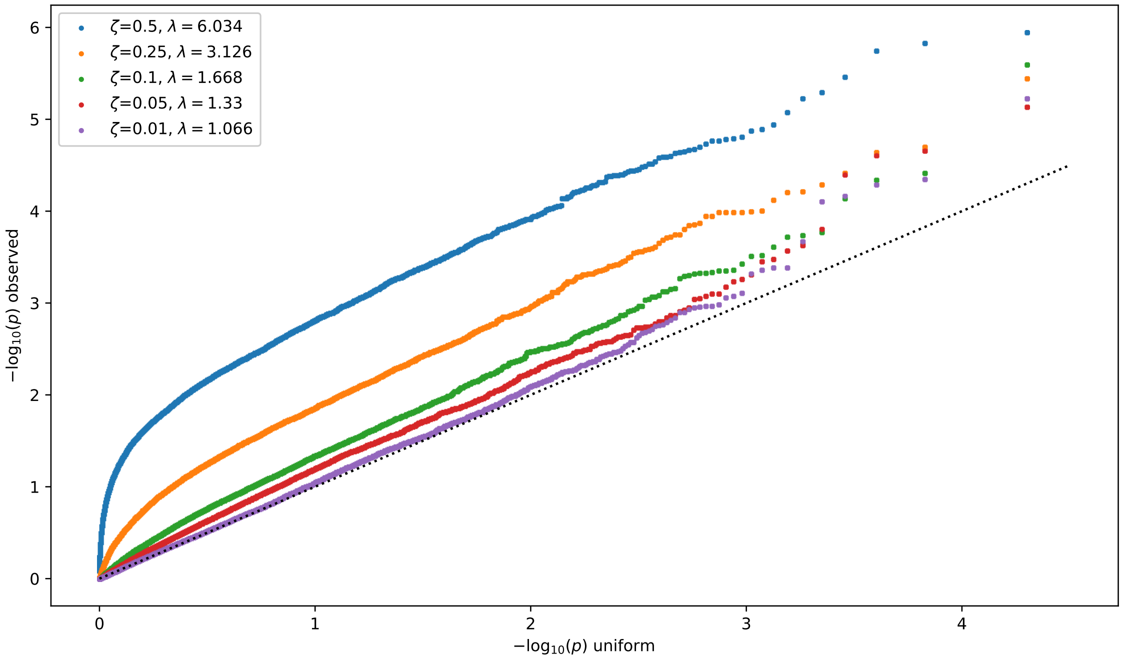

Fig 5: QQ plot for simulation of the index (20) without applied correction for ζ for various values of ζ. λ is defined as in Fig 3.

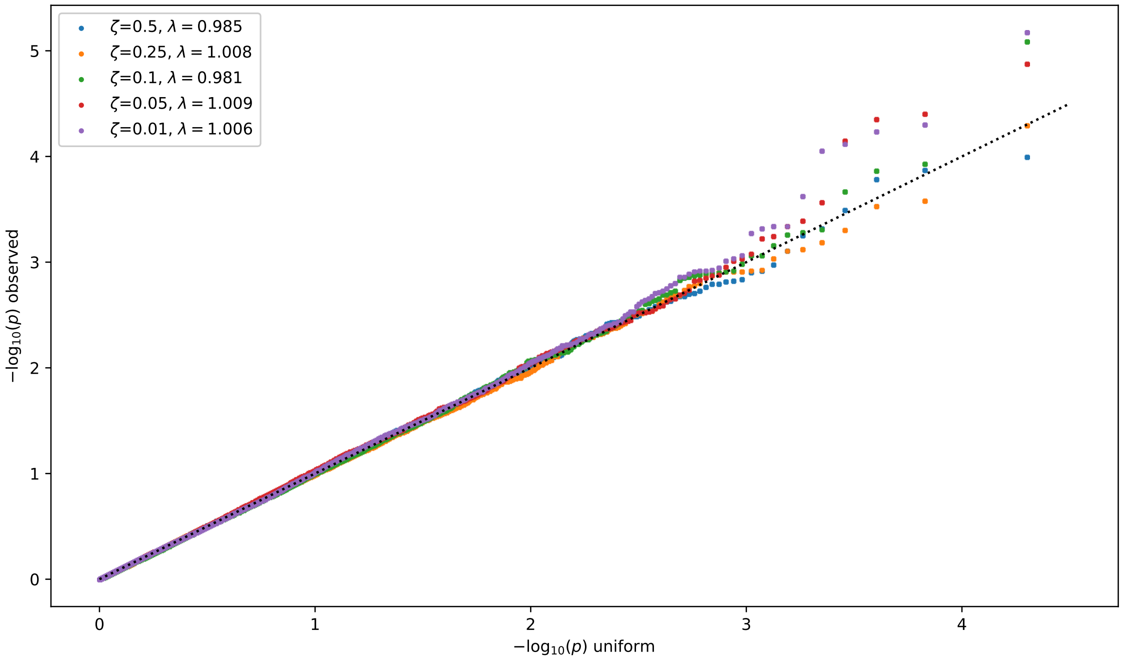

Fig 6: QQ plot for simulation of the index (20) with applied correction for ζ for same values of ζ as in Fig 5. λ is defined as in Fig 3. After applying the correction, the p-value calibration improved significantly and λ is close to one.
