# Supplementary material for: Cross-GWAS coherence test at the gene and pathway level
Source: PLoS Comput Biol. 2022 Sep 26;18(9):e1010517. doi: 10.1371/journal.pcbi.1010517 (PMC9536597; doi:10.1371/journal.pcbi.1010517)
Supplement: Attachment — Submitted filename: Rebuttal_ PCOMPBIOL-D-21-02179.pdf [file pcbi.1010517.s010.pdf]

Point-by-point response to reviewers

Reviewer #1: This manuscript introduces a new method for estimating gene-level joint genetic effects simultaneously from two traits profiled through a pair of GWAS. This new method is based on testing against a null hypothesis defined by the product-normal distribution. The approach is very neat and the experimental results provided in the manuscript show that it works very well, and it is implemented as an open-source Python package freely available on GitHub. I do not see caveats or shortcomings in the presented work and therefore I can only congratulate the authors for their thorough work and give them the following very minor suggestions.

We thank the reviewer for the positive comments.

1. I do not think that the first paragraph of the introduction is the right motivating example, because when one thinks of a Pearson correlation calculated from independently drawn samples, we typically associate those samples with subject individuals and not with SNPs, which are the unit that are later defined not to be independent when in close proximity (pg. 3, second paragraph).

We agree with the reviewer that our introductory paragraph was not ideal for the readership of *PLoS CompBio* and we decided to motivate our work directly within the context of GWAS to avoid the possible confusion mentioned by the reviewer. We have removed this paragraph and the first sentence of the second paragraph from the beginning of our introduction and now start with:

Genome-wide association studies (GWAS) correlate genotypes, most commonly single nucleotide polymorphisms (SNPs), with a phenotype of interest, both measured in the same study population. For human studies usually …

To highlight and better explain the fact that our statistic properly corrects for effects estimated from correlated SNPs (avoiding the term ‘sample’) we have revised the paragraph before the final one of our introduction:

Here, we propose the sum over the products between the effects of two traits for SNPs within a gene region as a simple measure for pleiotropy. For a single SNP, the test statistic is a simple product that is tested against the product-normal distribution, corresponding to a multiplicative meta-analysis, rather than an additive one like Fisher's. For multiple SNPs, our measure corresponds to the (non-centered) covariance between two sets of effect sizes. Importantly, we show that under the null hypothesis the corresponding test distribution can be expressed as a linear combination of χ^2 distributions, with a mixture of positive and negative coefficients. This holds even if the effect sizes are not independent of each other due to LD, \ie, if there exists a non-trivial covariance structure between the corresponding SNPs. The corresponding cumulative distribution function can be efficiently calculated with Davies' algorithm at high precision \cite{Davies1973,Davies1980}\revise{Citations added}. Thus, our method considers not only isolated significant SNPs, but all SNPs within the gene region to call a gene co-significant for two traits. Furthermore, using the notion of Mendelian

randomization, our statistic can be extended to test for a possible causal relationship between the two traits mediated by the tested gene.

In fact we also decided to change the title of our paper to focus more on the GWAS context, which now reads:

Cross-GWAS coherence test at the gene and pathway level

2. In page 4, when it says "Hence, even though the two traits share the same significant gene, they may not share the same functional mechanism", I think in the last bit you probably mean ".. they may not share the same genetic mechanism".

We agree and changed the expression as suggested.

3. In page 5 you mention Davies' algorityhm for the first time, please include also there the citation to the paper.

We thank the reviewer for spotting this and cited the reference at the position suggested. We also took this opportunity to cite another very relevant work of Davies, which we forgot to cite in the original submission.

4. In page 5, you define the distribution of the prodcut of two Gaussian random variables as "I ~ X(N, 1), with X being the variance-gamma distribution". However, X is a confusing choice of notation because first, it is more tipically used to denote a single numerical random variable and, second, you use it in page 26 to denote the genotype matrix. One straightforward solution could be to add VG as superscript to X.

We agree and changed our notation in the main text and supplement to VG(N,1).

5. Axis tickmarks and labels in figures 1, 2 and 4 to 10 are too small to be read comfortably.

We increased the size of figures such that the size of tick marks and labels are now more readable. We are happy to provide high resolution figures for the final typesetting, if needed.

6. In figure 2, please use panel letters instead of tagging "Top" and "Bottom".

We corrected the orientation of the figure to match the annotation. Of course, we are happy to annotate this and the other figures by letters, if preferred by the journal.

Reviewer #2: The study by Krefl & Bergmann describes a new method for detecting genes with coherent association signals for two traits. They devised a new statistical test and applied it to extract the genetic overlap between COVID-19 severity and other traits. The authors propose that the method could be extended to assess causality through a MR-type of analysis. There are merits in the algorithm and the concept could be useful for the field.

We thank the reviewer for the positive comments.

Major comments:

1- Introduction: Overall, there are general problems in introducing concepts of the algorithms searching for genetic correlation between traits, also when introducing LD blocks, which have many issues as they typically contain variants that are not in strong LD with the reminder.

We agree with the reviewer that methods searching for genetic correlations between traits in general have problems because there are residual dependencies between the local regions, even if one does LD decomposition. This is actually true for the classical local correlation algorithms like HESS etc. We would like to clarify, however, that our approach is quite different. Specifically, we do not attempt to decompose genetic correlation into local regions, but only test if the effects from two traits tend to be coherent in direction and magnitude over a set of SNPs in a given gene window. Our main contribution is to derive the null distribution for the test-statistic we propose to this end, even for correlated effect sizes (due to LD) and to show that its PDF can be computed fast and accurately. It is true, however, that there might be linked effects outside the gene-window that we miss, yet trying out different window sizes did not have any major impact on our results. We hope that this explanation and our revised introduction (see response to reviewer #1) provide clarifications.

2-Lines 173-178: This property is very interesting. However, the manuscript does not clearly explore if the approach is overly conservative. The question of reducing false positives is briefly assessed and discussed in the context of a simulation study, but it would be interesting to show additional data about conservativeness and further discuss.

We like to thank the reviewer for asking us to revisit this section. There was actually a small error in the corresponding original figure 1 as the Fisher p-values had been accidently calculated for 2 instead of 4 degrees of freedom. The corrected plot (shown below) shows more clearly the relevant property: Our method penalises divergence of p-values. Therefore, with increasing divergence, our method becomes more and more conservative. In contrast, for very similar p-values, our method becomes less stringent than Fisher's. This property is precisely what we need for our application, as we want to test for similarity of (ranked) effect sizes over a gene window: If the effect sizes are not very similar, we require stronger evidence (signal strength from one GWAS only) to count this as a co-signal.

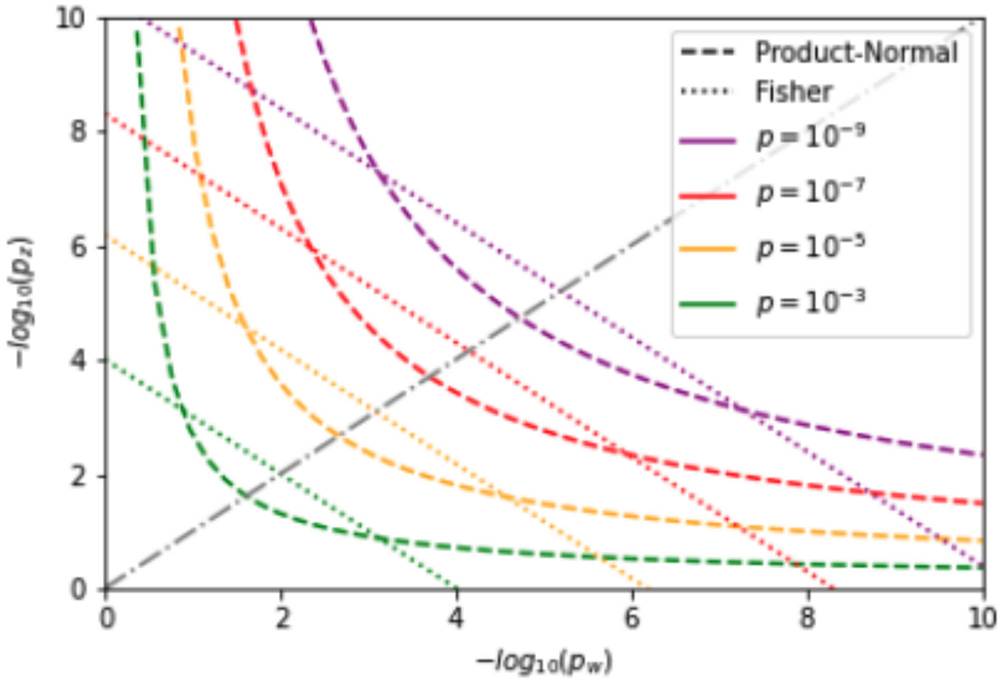

3-A comparison of results against existing methods of colocalization would be desirable, at least in the context of the main results with COVID-19.

> If the reviewer is referring to classical local correlation algorithms like HESS, we believe their goal is too different from ours (i.e. focusing on LD blocks rather than individual genes) to make any useful comparison (see also our response to the first comment). If the comparison is between additive (Fisher) and multiplicative (our method) signal integration, we believe that our Figure 1 now highlights the difference more clearly (see also our response to the second comment). If the reviewer knows of any other method that integrates signals from GWAS pairs at the level of genes we will be very happy to compare such a method to ours.

4-In the real GWAS settings, how is significance threshold calculated when cross-scored against medications? In most situations, the authors only considered the number of genes for the penalty and not the number of different medications being tested. This should be indicated and be considered to draw conclusions.

> We thank the reviewer for asking us to clarify our corrections for multiple hypotheses testing. We had only mentioned the corrections we applied in our figure legends but not in the main text. We have now added the relevant information. Specifically, we applied a Bonferroni correction accounting for both the number of tested genes *and* cross-tested traits, respectively. For the pathway enrichment test, we also correct now for the number of traits tested (six for the coherence test and seven for the ratio test).

5-What are the risks and potential biases of the results for cross-scoring across GWAS sharing individuals? This needs assessments or at least a discussion.

> We thank the reviewer for raising this point. In fact, we were able to address this question mathematically and work out and test the proper corrections for sample overlap. We added this work to our Supplement, which we summarise as follows in the main text:
>
> > *Nevertheless, even when co-analyzing data from GWAS with overlapping populations, it is possible to correct for the bias introduced by the phenotypic correlation (see Supplement for derivations and testing results).*

6-How would the test behave in situations where only one or a few SNPs from the gene are associated with strong p values? The example of COVID-19 for the two loci highlighted are not good examples of that because there are many variants associated in the two regions.

> We have clarified this point in the introduction as follows:
>
> > *For a single SNP, the test statistic is a simple product that is tested against the product-normal distribution, corresponding to a multiplicative meta-analysis, rather than an additive one like Fisher's.*

7-How is each gene defined to aggregate the p-values for the test? This is not explained and is a central part of the methods for it to be adaptable and reproducible by others. Besides, this approach has obvious limitations in the context of complex traits as many of the GWAS hits lie outside genes and would entail a difficulty for adding or linking their effects to distant genes. Please, discuss.

We now highlight in the main text that per default we consider a 50k window before and after the gene body and we refer to the Supplement to show that for a range of window sizes our results remain essentially unchanged. (Before we had only mentioned this under ‘Code availability’).

8-Overall the causation analysis of severe COVID-19 linked to vitamin D deficiency should be downplayed unless compared with MR approaches as a sanity check.

We reformulated the corresponding section of our manuscript as follows:

We found that Vitamin D carries two interesting hits suggesting causal pathways from Vitamin D concentration to severity of COVID-19, namely, the genes {\it KLC1} and {\it ZFYVE21}. The observed p_R-values are indicative of a potential causal flow from genetic predisposition for vitamin D concentration to severity of COVID-19, mediated via these genes.

9-What is the connection between higher likelihoods of M05B medication and severe COVID-19? If I interpreted this correctly (lines 267-268), the M05B medication likelihood should increase with age. Genetic risks for COVID-19 reduces with increasing age (as opposed to immune factors, comorbidities, etc). Thus, if both GWAS are referring to the same effect allele, we should be expecting an anti-coherence. Is that correct? Please, clarify this in the text as this is not obvious from the language used in the manuscript.

The COVID-19 GWAS has been performed by the original authors with age, age^2, age × sex as covariates. The medication GWAS also uses age as covariate. Therefore, age should not play a significant role in our analysis as age dependent effects should have been regressed out already. Rather, what we compare is the age-independent genetic predisposition / risk for the need to take M05B medication and the predisposition for severe COVID-19. What we observe, cf. figure 4 and 5, is that the effect alleles for significant genes are coherent in direction between M05B and severe Covid. That means that there is a shared genetic risk mediated via the significant genes *(CCR1,CCR3,LZTFL1).* We now clarify this as follows:

Note that, as usual, these GWAS have been performed with age as one of the covariates. Therefore, in our pair-wise analysis age-dependent effects are already regressed out and do not play a significant role in our analysis.

Minor comments:

English grammar needs to be improved.

We have asked a native speaker to read our manuscript resulting in a number of grammar improvements.

Abstract: Th1 to Th2 immune reaction. What does it mean reaction in this context? Clarify or modify the sentence

We changed our text from ‘reaction’ to ‘response’.

Better define the severe COVID-19 phenotype in the text. There are many and different comparisons (A1, A2, C) in that consortium and should be make clearer which analysis is being used. It is not described in the main text nor in the SM.

> The particular data used had been stated already in textual form in the main text (eur population, very severe covid, without UKBB) and the particular file had been already stated in the "data availability" statement ("A2_ALL_eur_leave_ukbb_23andme, release 7. Jan. 2021"). However, to make this more clear, we also added "A2_ALL_eur_leave_ukbb" to the main text.

Figure 5 and other Manhattan plots: please, add their corresponding QQ plots and lambdas as control. In the legend, it is not clear if correlation is referring to R2 and if the data for its calculation is for all the 1KG reference panel or for the particular population composition involved in the GWAS results. Clarify.

> We added QQ-plots and lambda estimates to our supplement. All lambdas are in the commonly accepted range of < 1.1. We added a sentence to the main text pointing out that we use the European sub-population of the 1KG high coverage release.

Figure 6 (also for figures 4 and 5 of the SM): Use of alternative colors for this figure of logP by position is desirable for better distinguishing positive and negative associations. Green and orange are not easy to distinguish.

> We changed the colour scheme of these figures. In particular, a printout of the figures now has sufficient contrast.

Table 1: A clarification of the type of drugs the first column refers to will be helpful for the reader.

> We had stated the types of drugs for the drug codes most relevant to our results, but for the reader's convenience, we took up the reviewer's suggestion and added a table to the supplement listing the type of drugs for all the 23 investigated medications.
